# Dendrimers for Drug Delivery: Where Do We Stand in 2023?

**DOI:** 10.3390/pharmaceutics15122740

**Published:** 2023-12-07

**Authors:** Evgeny K. Apartsin

**Affiliations:** Univ. Bordeaux, CNRS, Bordeaux INP, CBMN, UMR 5248, F-33600 Pessac, France; evgeny.apartsin@u-bordeaux.fr

Dendrimers are highly symmetric, hyperbranched macromolecules consisting of repeating structural units. Owing to their peculiar synthesis, the structure of dendrimers is perfectly defined and highly reproducible. An isolated branch of a dendrimer is called a “dendron”. By choosing the structural elements of dendrimers, it is possible to modulate their physicochemical properties, adjust the molecular weight and the density of functional groups, and modify dendrimers with different functionalities. The synthetic flexibility permits an optimization of their structure according to a given task.

Dendrimers and dendrons are known for their so-called “dendritic effect”, i.e., a dramatic increase in efficiency when using a dendrimer compared to a monomer, increasing with each generation. The dendritic effect is influenced by the nature and size of the core as well as the cooperativity of functional groups on the periphery. The dendritic effect-enhanced multivalency and uniformity of structure are major differences between dendrimers and hyperbranched polymers. Frequently, they define the dendrimer properties and behavior in different processes at the nanoscale and at the nano-bio interface.

Dendrimer science started with the burst of synthetic chemistry methods several decades ago, and a great variety of dendrimer architecture has been developed, with hundreds of species synthesized. However, the field will become strongly application-oriented by 2023. Importantly, dendrimers’ features mentioned above explain great interest to them in biology and medicine. The recent success of dendrimer-based medical formulations, for instance, Vivagel^®^ approved by the US FDA, proves the significant potential of dendrimers in biomedical applications.

Today, there will be no surprise for readers claiming that drug delivery is still challenging in many aspects, such as the development of safe and efficient vaccines (including DNA and mRNA vaccines), antitumor drug-loaded supramolecular constructions, nanoformulations for treating neurodegenerative disorders, bacterial and viral infections, etc. Using dendrimers, either as drug carriers or as drugs per se can provide new opportunities to tackle pathologies that are unreachable or inefficiently treated with the existing formulations.

This Special Issue of *Pharmaceutics* entitled “Dendrimers for drug delivery” assembles both reviews and research papers focused on distinct aspects of dendrimer applications in healthcare and written by recognized experts in the field. The reviews highlight the use of dendrimers in the diagnostics and therapy of Alzheimer’s disease [1], the development of dendrimer-based formulations for DNA and RNA vaccines [2], and dendrimer-mediated cancer nanomedicine [3]. The latter review is particularly interesting as it summarizes the activities of a huge international consortium, NANO2CLINIC, dealing with cancer nanomedicine oriented on the application of dendritic molecules.

Research papers published in the Special Issue cover applications of dendrimer-based formulations in therapy and imaging. M. Neugebauer et al. describe a PCR-based method to assess the quantity and integrity of small interfering RNAs (siRNAs) in polyelectrolyte complexes with cationic dendrimers (i.e., dendriplexes). Having a picomolar sensitivity, this method is promising for controlling the stability of dendrimer-oligonucleotide formulations [4].

N. Knauer et al. reported a study of the effects of dendriplexes containing immunomodulatory microRNAs on non-activated immunocompetent cells. Dendriplexes of miR-155 or its synthetic inhibitor with polycationic dendrimers affected the expression of HLA-DR on T-cells and PD-1 expression on T and B lymphocytes. The treatment with dendriplexes also affected the production of IL-4 and IL-10, but not the production of perforin and granzyme B [5]. The interaction of dendrimer-based carriers with immune cells was also studied in the paper of H. Shiba et al. The authors have synthesized a series of PAMAM dendrimers modified with 1,2-cyclohexanedicarboxylic acid and phenylalanine moieties grafted in distinct order and to a different extent. Species bearing phenylalanine at more than half of the termini exhibited a higher association with T cells and other immune cells, with the highest affinity being reported to occur at 75% grafting. The robustness of the functionalized dendrimer as a drug vehicle was demonstrated by the successful delivery of a model drug, protoporphyrin IX (PpIX) into T cells [6]. These findings can be important for the development of dendrimer-based tools for immunotherapy.

M. Szota and B. Jachimska studied the biophysical parameters of doxorubicin complexation with PAMAM dendrimers under alkaline conditions. They have shown that at a pH 9.0–10.0, the chemodrug and the dendrimer form stable complexes, where one dendrimer molecule can bond 1 to 10 molecules of doxorubicin. The complexes also possess high fluorescence intensity, which can be useful for theranostics [7].

Dendrimers can also be a carrier for macromolecular imaging agents. J. Hersh et al. engineered a fusion protein consisting of an EGFR-specific antibody and a Gaussia luciferase bioluminescent protein. The complexation with a G5-PAMAM dendrimer improved the protein stability in vivo and increased signal strength. The bioluminescent complexes could delineate the tumor shape, identify multiple masses, and locate metastases in vivo in pancreatic cancer xenograft mice [8]. These results prove the versatility of dendrimer-mediated delivery of bioluminescent proteins as a way to improve in vivo bioluminescent imaging.

As a Guest Editor, I would like to sincerely thank the contributors and the reviewers who have done an amazing collaborative job to issue high-quality papers of undoubted interest.
